# DUSP5 functions as a feedback regulator of TNFα-induced ERK1/2 dephosphorylation and inflammatory gene expression in adipocytes

**DOI:** 10.1038/s41598-017-12861-y

**Published:** 2017-10-10

**Authors:** Justine S. Habibian, Mitra Jefic, Rushita A. Bagchi, Robert H. Lane, Robert A. McKnight, Timothy A. McKinsey, Ron F. Morrison, Bradley S. Ferguson

**Affiliations:** 1 University of Nevada, Department of Agriculture, Nutrition, and Veterinary Sciences, Reno, Reno, Nevada 89557 USA; 2 University of Colorado Denver-Anschutz Medical Campus, Department of Medicine, Division of Cardiology and Consortium for Fibrosis Research & Translation, Aurora, Colorado, 80045 USA; 3 Medical College of Wisconsin, Department of Pediatrics, Milwaukee, Wisconsin 53226 USA; 4 University of Utah, Department of Pediatrics, Salt Lake City, Utah 84108 USA; 5 University of North Carolina Greensboro, Department of Nutrition, Greensboro, North Carolina 27412 USA

## Abstract

Adipose tissue inflammation is a central pathological element that regulates obesity-mediated insulin resistance and type II diabetes. Evidence demonstrates that extracellular signal-regulated kinase (ERK 1/2) activation (i.e. phosphorylation) links tumor necrosis factor α (TNFα) to pro-inflammatory gene expression in the nucleus. Dual specificity phosphatases (DUSPs) inactivate ERK 1/2 through dephosphorylation and can thus inhibit inflammatory gene expression. We report that DUSP5, an ERK1/2 phosphatase, was induced in epididymal white adipose tissue (WAT) in response to diet-induced obesity. Moreover, DUSP5 mRNA expression increased during obesity development concomitant to increases in TNFα expression. Consistent with *in vivo* findings, DUSP5 mRNA expression increased in adipocytes in response to TNFα, parallel with ERK1/2 dephosphorylation. Genetic loss of DUSP5 exacerbated TNFα-mediated ERK 1/2 signaling in 3T3-L1 adipocytes and in adipose tissue of mice. Furthermore, inhibition of ERK 1/2 and c-Jun N terminal kinase (JNK) signaling attenuated TNFα-induced DUSP5 expression. These data suggest that DUSP5 functions in the feedback inhibition of ERK1/2 signaling in response to TNFα, which resulted in increased inflammatory gene expression. Thus, DUSP5 potentially acts as an endogenous regulator of adipose tissue inflammation; although its role in obesity-mediated inflammation and insulin signaling remains unclear.

## Introduction

Obesity is a global epidemic and major public health concern, in which obesity increases the risk of chronic illnesses such as; cardiovascular disease, stroke and type II diabetes^1^. Chronic, low-grade inflammation plays a causal role in the loss of insulin sensitivity of adipose tissue; adipose tissue inflammation and dysfunction link obesity to the pathogenesis of type II diabetes^2,3^. A key event in obesity is characterized by macrophage infiltration in adipose tissue, which triggers increased secretion of pro-inflammatory adipokines such as monocyte chemoattractant protein-1 (MCP-1) and tumor necrosis factor α (TNFα). Secretion of MCP-1 further attracts macrophages to adipose tissue, exacerbating pro-inflammatory adipokine expression that results in chronic inflammation and systemic insulin resistance^3^.

Mitogen activated protein kinases (MAPKs) play a central role in the regulation of adipose tissue inflammation and insulin signaling^4–7^. Extracellular signal-regulated protein kinase (ERK1/2), c-Jun N-terminal kinase (JNK) and p38 comprise the classical MAPKs and are involved in obesity-induced inflammation and insulin resistance^7^. For example, mice deficient in ERK1 are protected against diet-induced obesity and insulin resistance^8^. Substantial evidence demonstrates that TNFα perpetuates chronic inflammation leading to insulin resistance, as ablation of TNFα restores insulin sensitivity^9–11^. MAPK signaling links TNFα to cytosolic inhibition of insulin signaling via IRS-1 regulation and nuclear expression of pro-inflammatory adipokines^5,7,12^.

While it is well recognized that MAPKs are activated by phosphorylation of the conserved threonine and tyrosine residues within the T-X-Y motif in the activation loop by upstream MAPK kinases, less is known about their deactivation by phosphatases in the regulation of adipose inflammation^6,13,14^. Dual specificity phosphatases (DUSPs) constitute a subclass of protein tyrosine phosphatases that specifically dephosphorylate (i.e. deactivate) MAPKs. DUSPs are inducible protein phosphatases that have been shown to regulate cellular inflammation and more recently have been shown to play a role in obesity and insulin resistance, in particular through the regulation of MAPK deactivation^14–16^.

In this report, we show that TNFα-mediated phosphorylation of ERK1/2 and JNK stimulated expression of the ERK-specific phosphatase, dual-specificity phosphatase 5 (DUSP5) resulting in feedback inhibition of ERK signaling and ERK-dependent inflammatory gene expression in adipocytes. Consistent with these findings *in vitro*, DUSP5 deletion in mice exacerbated TNFα-mediated ERK1/2 phosphorylation and inflammatory protein expression in adipose tissue. Our findings highlight the prospective role for DUSP5 in the regulation of adipocyte inflammation and suggest that phosphatases offer potential therapeutic targets to regulate MAPK-dependent inflammation.

## Results

### DUSP5 positively correlates with TNFα expression in adipose tissue of diet-induced obese mice

Adipose tissue is a predominate site for the development of obesity-associated inflammation leading to systemic metabolic dysfunction^10,12^. As such, we examined DUSP5 and TNFα gene expression in response to body weight gain in mice over time. For these studies, C57BL/6 J male mice were given ad libitum access to a high fat diet (HFD; 60% kcal) or control diet (CD) starting at six weeks of age. Studies were conducted at 18 wks (stage I) and 24 wks (stage II) of age, representing 12 wks and 18 wks of specialized diet, respectively. As shown in Table 1, 18 wk and 24 wk old mice fed a HFD presented with a 13% and 26% increase in body weight, relative to lean mice fed the CD.

**Table 1:** Anthropometrics of mice in this study.

Stage	Age	Final BW (g)		% ∆
*diet-induced obesity*		**LFD**	**HFD**	
I	18 wk	30.8	34.7	13%
II	24 wk	32.2	40.6	26%

Percent difference in body weight (BW) between lean and obese mice (% ∆).

**Figure 1:**
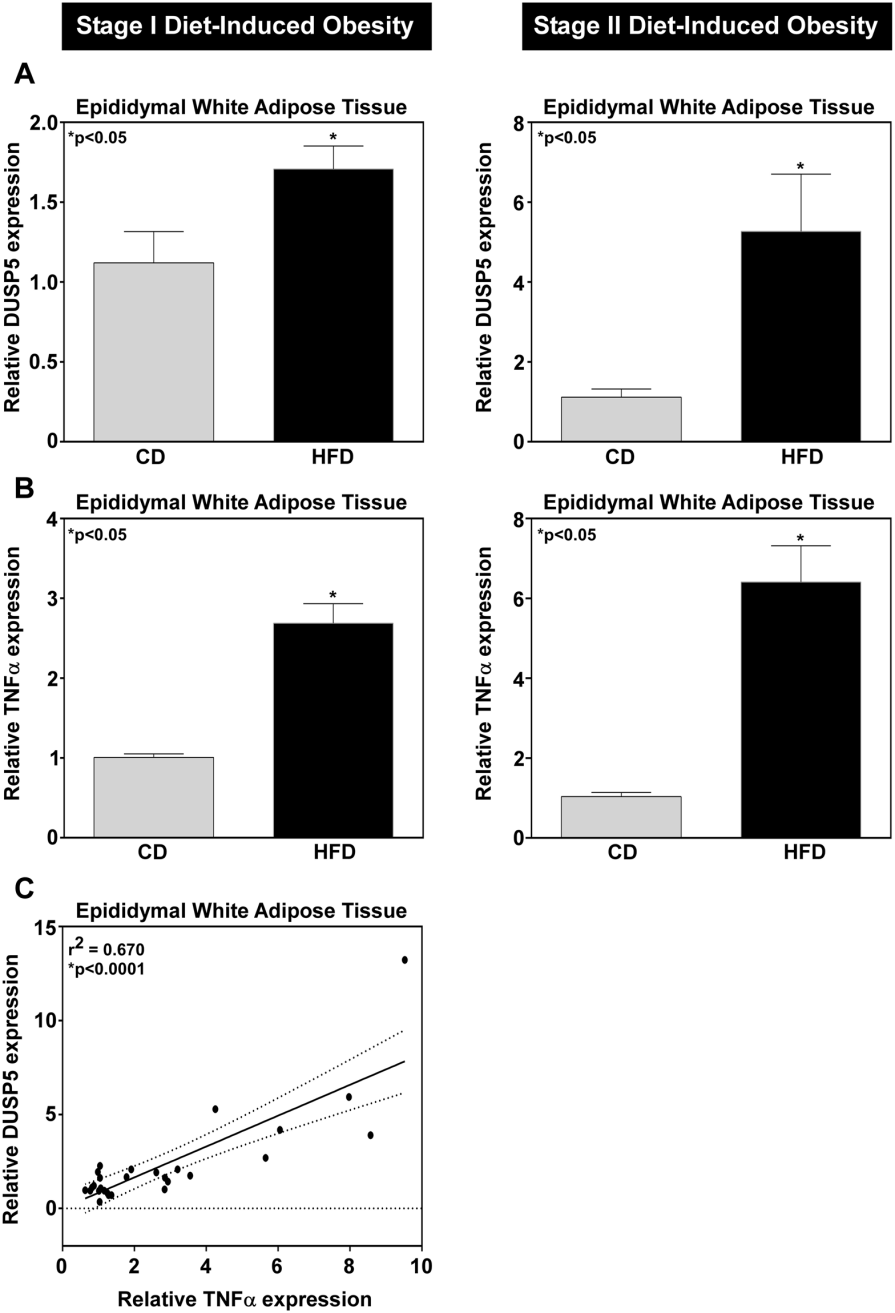
Increased DUSP5 mRNA expression correlates to increased obesity-mediated TNFα inflammation. RNA was isolated from white adipose tissue (WAT) and (**A**) DUSP5 and (**B**) TNFα mRNA expression assessed via qPCR. As the degree of obesity increased from stage I (12 wk HFD) to stage II (18 wk HFD), (**C**) TNFα mRNA expression increased; this correlated with increased DUSP5 message. Student’s t-test with Welch’s correction and Pearson Correlation were used to determine significance p < 0.05.

To assess the inflammatory status at each stage, relative mRNA expression was determined for TNFα, a critical inflammatory marker in the development of chronic adipose tissue inflammation^10,12^. TNFα gene expression was increased in adipose tissue from epididymal depots of diet-induced obese (DIO) mice, with a progressive increase in TNFα abundance correlating with increased obesity (Fig. 1B). DUSP5 gene expression proportionally increased with increased obesity (Fig. 1A) and this positively correlated to an increase in TNFα (Fig. 1C).

### TNFα regulates DUSP5 expression in adipocytes

**Figure 2:**
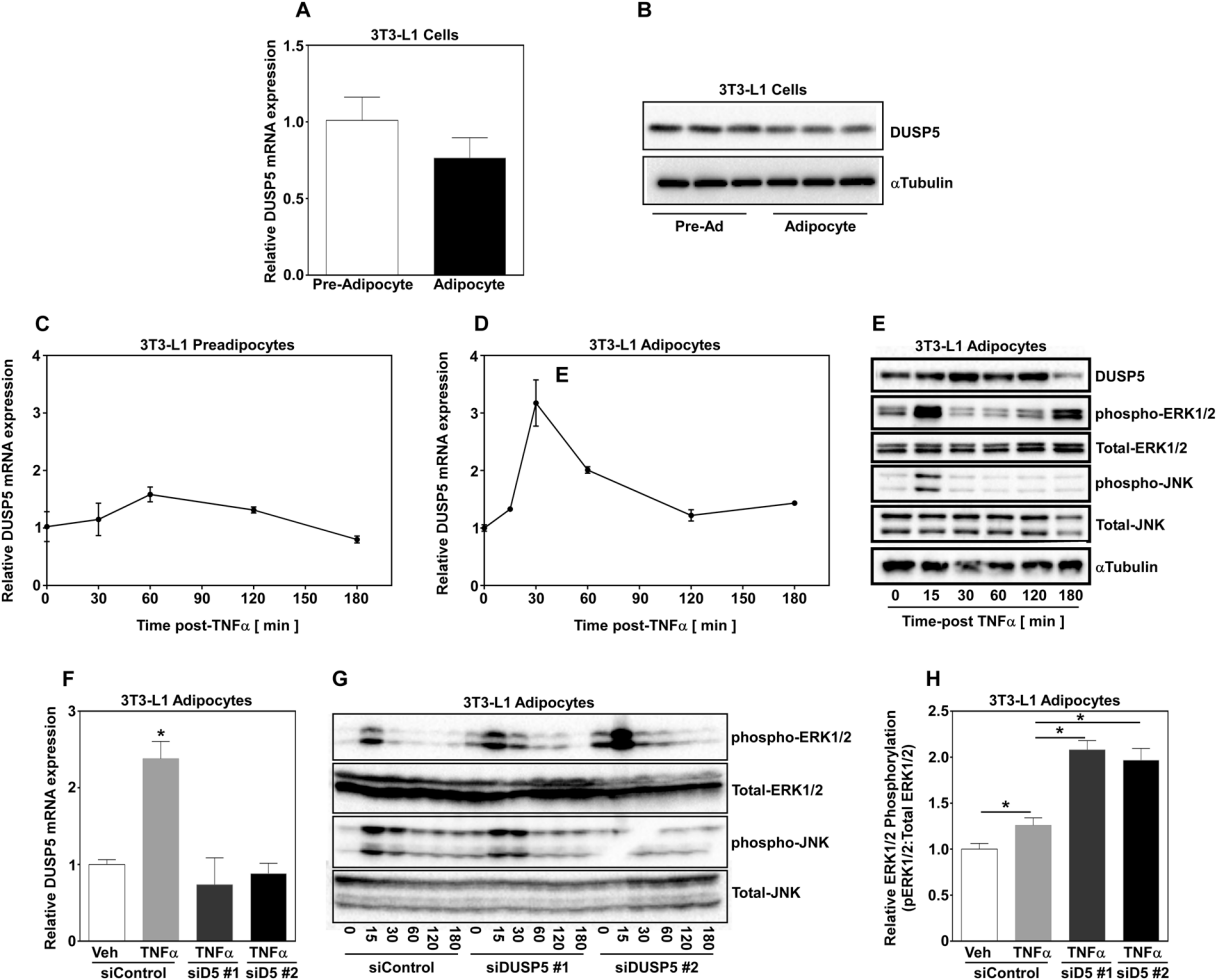
DUSP5 expression is essential for the regulation of TNFα-induced ERK 1/2 phosphorylation. (**A**) RNA was isolated from 3T3-L1 pre-adipocytes and differentiated adipocytes and (**A**) DUSP5 mRNA and (**B**) protein expression was assessed via qPCR and immunoblotting, respectively. Pre-adipocytes and adipocytes were stimulated in parallel with TNFα (100 pM) and RNA isolated over time post-treatment prior to examination of DUSP5 mRNA expression via qPCR in (**C**) preadipocytes and (**D**) differentiated adipocytes. (**E**) 3T3-L1 adipocytes were stimulated without TNFα (100pM) and protein harvested over time. Whole cell lysate was subjected to immunoblotting for the detection of DUSP5, phospho-ERK and JNK as well as total ERK and JNK. Adipocytes were transiently transfected with small, non-targeting control (100 nM) siRNAs or siRNAs (100 nM) targeting independent regions of the DUSP5 gene for 48 hrs prior to stimulation with TNFα. (**F**) RNA was isolated 30 min post-treatment and qPCR used to examine DUSP5 expression. (**G**) Whole cell lysate was collected over time post-TNFα stimulation and immunoblotting used to assess phospho-ERK and JNK as well as total ERK and JNK. (**H**) Adipocytes (n = 3/group) were transiently transfected with small, non-targeting control (100 nM) siRNAs or siRNAs (100 nM) targeting independent regions of the DUSP5 gene for 48 hrs prior to stimulation with TNFα. Protein was collected 15 minutes post-TNFα stimulation and immunoblotted for phospho- and total-ERK. Image J software was used to quantify ERK phosphorylation to total ERK expression and one-way ANOVA with Tukey’s post-hoc analysis used to determine significance p < 0.05.

Adipose tissue is predominantly composed of stromal vascular cells that contain preadipocytes and mature adipocytes. Therefore, we examined DUSP5 mRNA expression in preadipocytes and adipocytes. The 3T3-L1 preadipocyte cell line can be stimulated to differentiate into mature adipocytes, with greater than 90% conversion to mature fat cells. We found that DUSP5 mRNA was expressed to similar levels in preadipocytes and differentiated mature day 8 adipocytes (Fig. 2A); protein expression mirrored mRNA expression (Fig. 2B; Supplemental Fig. 1A). Based on these results, and since TNFα expression positively correlated with DUSP5 expression *in vivo*, we next examined DUSP5 mRNA expression in 3T3-L1 preadipocytes and differentiated adipocytes stimulated with 100pM TNFα. This dose of TNFα has previously been shown to suppress insulin signaling and closely approximates TNFα concentrations in obese humans and animals^9,10^. Preadipocytes and mature adipocytes were stimulated in parallel with 100pM TNFα and RNA collected over time post-TNFα stimulation prior to qPCR analysis for DUSP5 gene expression. TNFα failed to stimulate DUSP5 mRNA expression in preadipocytes (Fig. 2C). In contrast, 100pM TNFα transiently (<1hr) increased DUSP5 mRNA expression in mature adipocytes (Fig. 2D).

DUSP5 is an inducible ERK-specific phosphatase^17–20^. Therefore, we examined ERK and JNK phosphorylation in mature 3T3-L1 adipocytes stimulated with 100pM TNFα. Cells were lysed for protein over time and immunoblotted with antibodies against total and phosphorylated ERK and JNK. TNFα-induced phosphorylation of ERK and JNK, which peaked at 15 minutes. This was followed by rapid dephosphorylation by 30 minutes (Fig. 2E; Supplemental Fig. 1B). DUSP5 mRNA (Fig. 2D) and protein expression (Fig. 2E; Supplemental Fig. 1B) increased concomitant to ERK dephosphorylation, suggesting that ERK phosphorylation is inversely proportional to DUSP5 expression.

### DUSP5 functions to dephosphorylate ERK1/2 in adipocytes in response to TNFα

Based on findings above, we postulated that DUSP5 regulates ERK dephosphorylation in adipocytes in response to TNFα. To test this postulate, adipocytes were transiently transfected with short interfering RNAs (siRNAs) targeting two different regions of the DUSP5 gene (siDUSP5) for 48 hrs prior to stimulation with 100 pM TNFα. A non-targeting control siRNA (siControl) was also transiently transfected under the same conditions. Total RNA was collected 30 minutes post TNFα stimulation and whole cell lysates harvested over time post-TNFα and analyzed for relative DUSP5 mRNA abundance as well as total and phosphorylated ERK and JNK. Targeted siRNA significantly suppressed TNFα-induced DUSP5 gene expression compared to siControl (Fig. 2F). Transfection with non-targeting siControl did not affect TNFα-induced DUSP5 expression (Fig. 2F). Loss of DUSP5 gene expression via siRNA was mirrored by prolonged ERK phosphorylation over time (Fig. 2G; Supplemental Fig. 1C), with a significant increase in the magnitude of ERK phosphorylation demonstrated 30 minutes post-TNFα stimulation (Fig. 2H). While loss of DUSP5 function exacerbated ERK phosphorylation (Fig. 2H), JNK phosphorylation remained unchanged. Lastly, transient transfection with siRNAs did not impact total ERK or JNK protein expression, demonstrating that DUSP5 specifically targeted ERK for dephosphorylation in adipocytes.

### DUSP5 regulates ERK-dependent inflammatory gene expression in adipocytes

**Figure 3:**
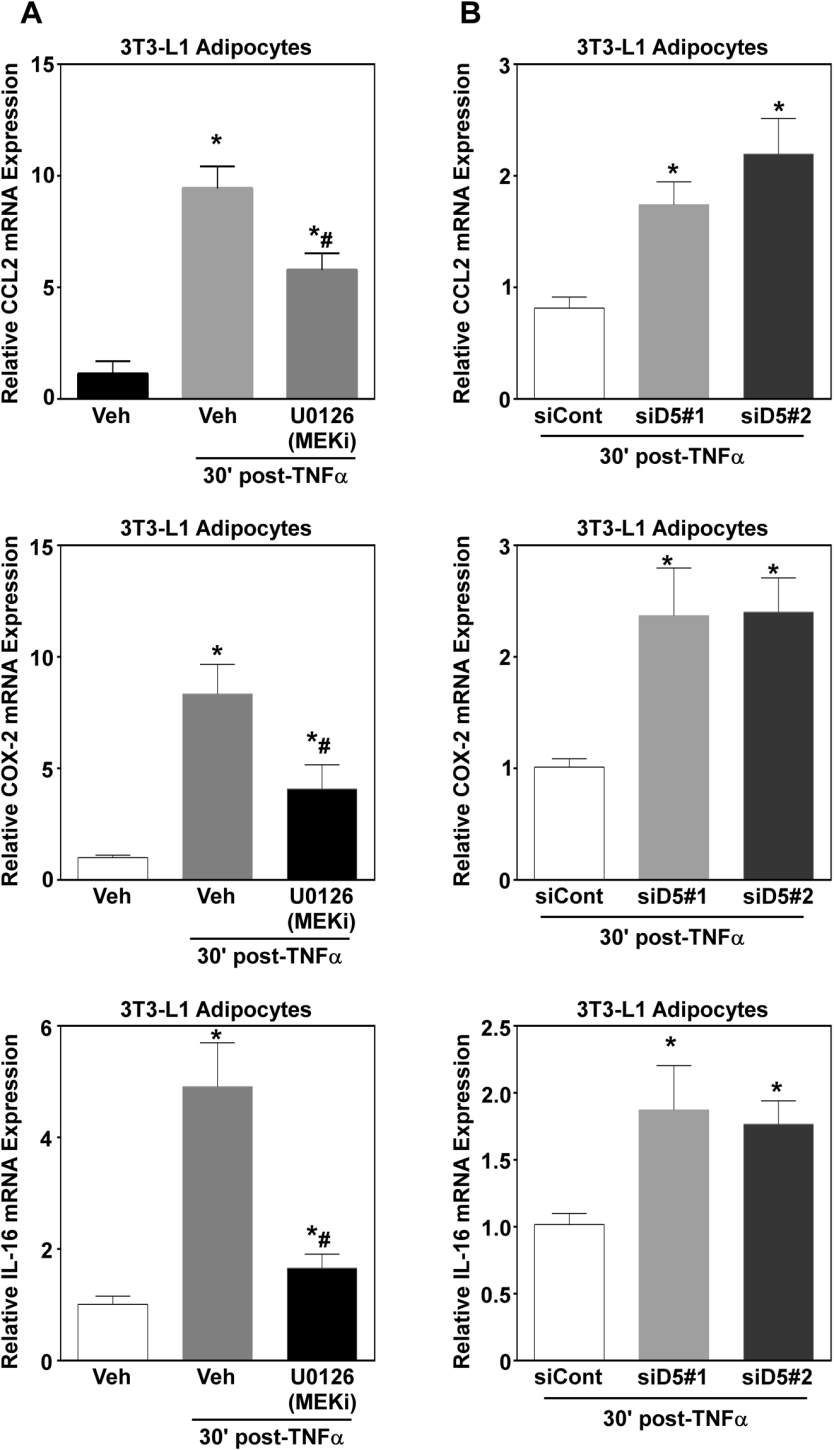
DUSP5 regulates ERK1/2-dependent inflammatory gene expression in adipocytes. (**A**) Adipocytes were pretreated (30 min) with the MEK1/2 inhibitor U0126 prior to stimulation with vehicle (H_2_0) or TNFα (100 pM). RNA was isolated 30 min post-TNFα stimulation and qPCR used to assess inflammatory gene expression of CCL2, COX-2, and IL-16. (**B**) Adipocytes (n = 3/group) were transiently transfected with small, non-targeting control (100 nM) siRNAs or siRNAs (100 nM) targeting independent regions of the DUSP5 gene for 48 hrs prior to stimulation with TNFα. RNA was isolated 30 min post-TNFα stimulation and assessed for CCL2, COX-2 and IL-16 mRNA expression. Statistical analysis was completed by ANOVA followed by Tukey’s post-hoc using GraphPad Prism software to determine significance p < 0.05.

Data presented above demonstrate that DUSP5 regulates ERK dephosphorylation. To determine if changes in ERK phosphorylation resulted in changes in adipocyte function, we next examined pro-inflammatory gene expression. Previous screening efforts from our lab (data not published), identified nuclear factor kappa B- and MAPK-dependent inflammatory genes. We report that TNFα stimulation resulted in a significant increase in inflammatory gene expression of C-C motif chemokine ligand 2/monocyte chemoattractant protein-1 (CCL2/MCP-1), cyclooxygenase-2 (Cox-2) and interleukin-16 (IL-16) (Fig. 3A). To determine a role for ERK activity regarding changes in gene expression, adipocytes were pretreated with U0126, a potent and selective inhibitor of the upstream ERK kinase, MEK, for 30 minutes prior to stimulation with TNFα. Total RNA was harvested after 1 hr post-TNFα stimulation. TNFα-induced CCL2, Cox-2 and IL-16 was significantly suppressed by ERK inhibition (Fig. 3A).

Based on findings above, we postulated that loss of DUSP5 would increase expression of ERK-dependent pro-inflammatory genes. To test this, adipocytes were transfected with two independent siRNAs targeting DUSP5 for 48 hrs prior to stimulation with TNFα. Knockdown of DUSP5 significantly exacerbated CCL2, COX-2 and IL-16 compared to siControl in response to TNFα stimulation; demonstrating biological significance for DUSP5-mediated regulation of ERK phosphorylation.

### ERK and JNK activity are necessary for TNFα-induced DUSP5 gene expression

**Figure 4:**
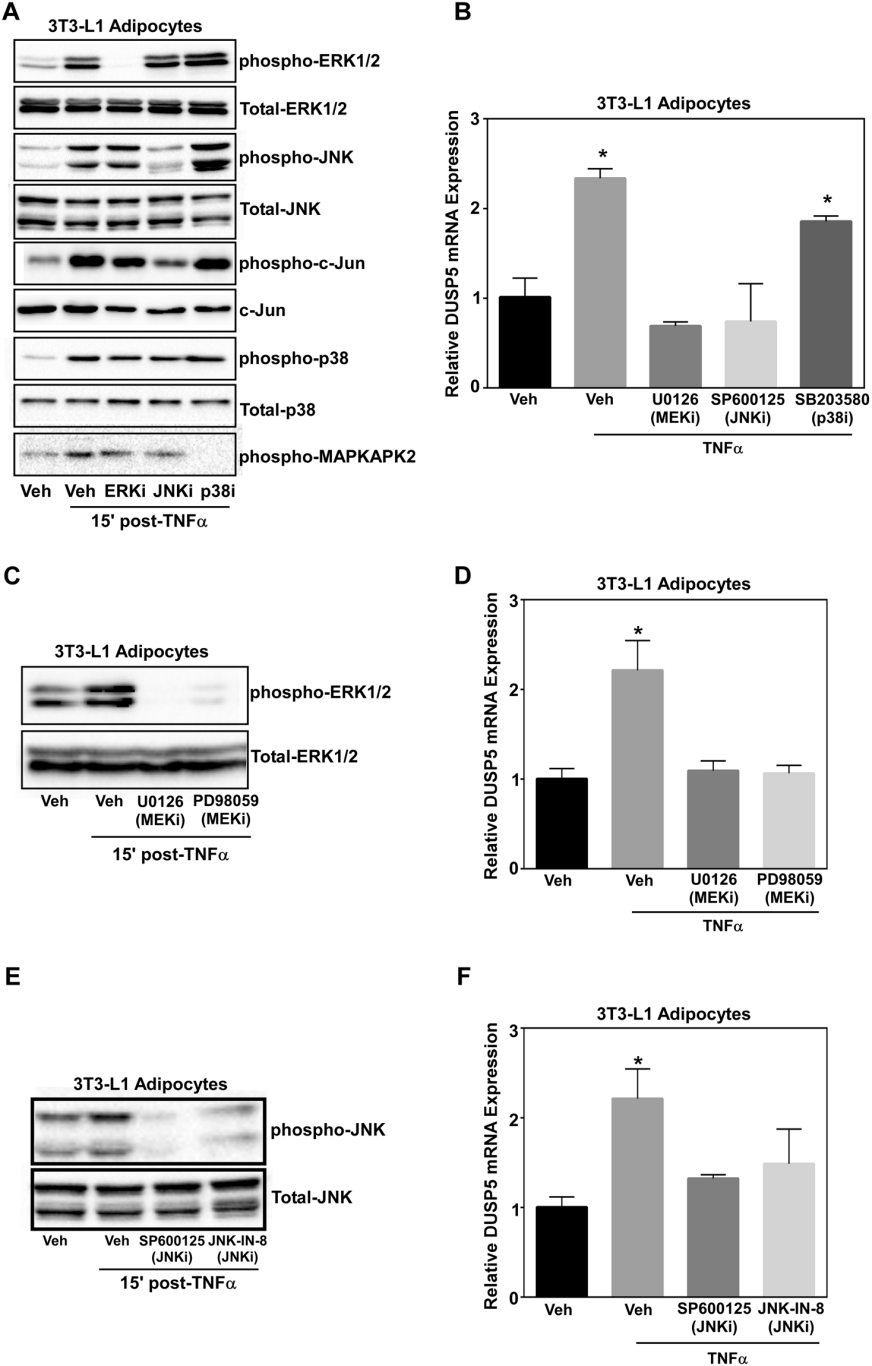
ERK and JNK activity are necessary for TNFα-induced DUSP5 gene expression. Adipocytes were pre-treated with the MEK inhibitor (U0126; ERKi)), JNK inhibitor (SP600125; JNKi) or p38 inhibitor (SB203580; p38i) for 30 min prior to stimulation with 100 pM TNFα. Protein was collected 15 min and RNA isolated 30 min post-TNFα stimulation and (**A**) immunoblotted for phospho-ERK, JNK, p38, c-Jun and MAPKAPK2 as well as total ERK, JNK, p38 and c-Jun. (**B**) DUSP5 mRNA expression was assessed via qPCR. Adipocytes were pre-treated with two independent MEK inhibitors U0126 and PD98059 for 30 min prior to stimulation with 100 pM TNFα. Protein was collected 15 min and RNA isolated 30 min post-TNFα stimulation and (**C**) immunoblotted for phospho-ERK as well as total ERK. (**D**) DUSP5 mRNA expression was assessed via qPCR. Adipocytes were pre-treated with the JNK inhibitors SP600125 or JNK-IN-8 for 30 min prior to stimulation with 100 pM TNFα. (**E**) Protein was collected 15 min post-TNFα stimulation and immunoblotted for phospho-JNK and total JNK. (**F**) RNA was isolated 30 min post-TNFα and qPCR used to examine DUSP5 mRNA expression. ANOVA followed by Tukey’s post-hoc using GraphPad Prism software was used to determine significance p < 0.05.

It has previously been reported that ERK regulates DUSP5 expression creating a feedback loop^21^. To determine if MAPKs regulate TNFα-induced DUSP5 gene expression, adipocytes were pretreated with U0126, SP600125, and SB203850, representing inhibitors of ERK, JNK, and p38, respectively, for 30 minutes prior to stimulation with TNFα. RNA was harvested 30 minutes post-TNFα stimulation, while whole cell lysates were collected 15 minutes post-TNFα and immunoblotted for total and phosphorylated ERK, JNK and p38. ERK phosphorylation was inhibited when cells were treated with the MEK inhibitor U0126 (Fig. 4A). Of note, JNK phosphorylation was inhibited with treatment of SP600125 (Fig. 4A). This is interesting as the JNK and p38 inhibitor act as ATP-competitive inhibitors and thus are thought to primarily interfere with downstream phosphorylation. Indeed, inhibition of JNK and p38 with SP600125 and SB203850, led to attenuation of c-Jun and MAPKAPK2 phosphorylation, respective downstream targets of JNK and p38 (Fig. 4A). Inhibition of both ERK and JNK significantly suppressed DUSP5 gene expression (Fig. 4B).

To validate these findings, we used two independent inhibitors of the ERK and JNK pathway. Adipocytes were pretreated with U0126 and PD98059 to inhibit ERK as well as SP600125 and JNK-IN-8 to inhibit JNK for 30 minutes prior to TNFα stimulation. TNFα-induced ERK phosphorylation was completely blocked with both ERK inhibitors (Fig. 4C). Moreover, inhibition of ERK significantly blocked DUSP5 expression in which gene expression was reduced back to vehicle treated levels (Fig. 4D). Similarly, TNFα-mediated JNK phosphorylation was attenuated with SP600125 and JNK-IN-8 (Fig. 4E). Consistent with these findings, TNFα-induced DUSP5 gene expression was significantly attenuated in the presence of JNK inhibition (Fig. 4F). While it should be noted that we observed no cross talk with the JNK or ERK inhibitors SP600125 and U0126 on MAPK signaling, we did not address actions of these inhibitors regarding other signaling pathways. Collectively, however, these data suggest that JNK and ERK regulate DUSP5 gene expression. For all MAPK inhibitor experiments, complete blots with molecular weight markers are shown (Supplemental Fig. 2).

### DUSP5 regulates ERK dephosphorylation and inflammation ***in vivo***

**Figure 5:**
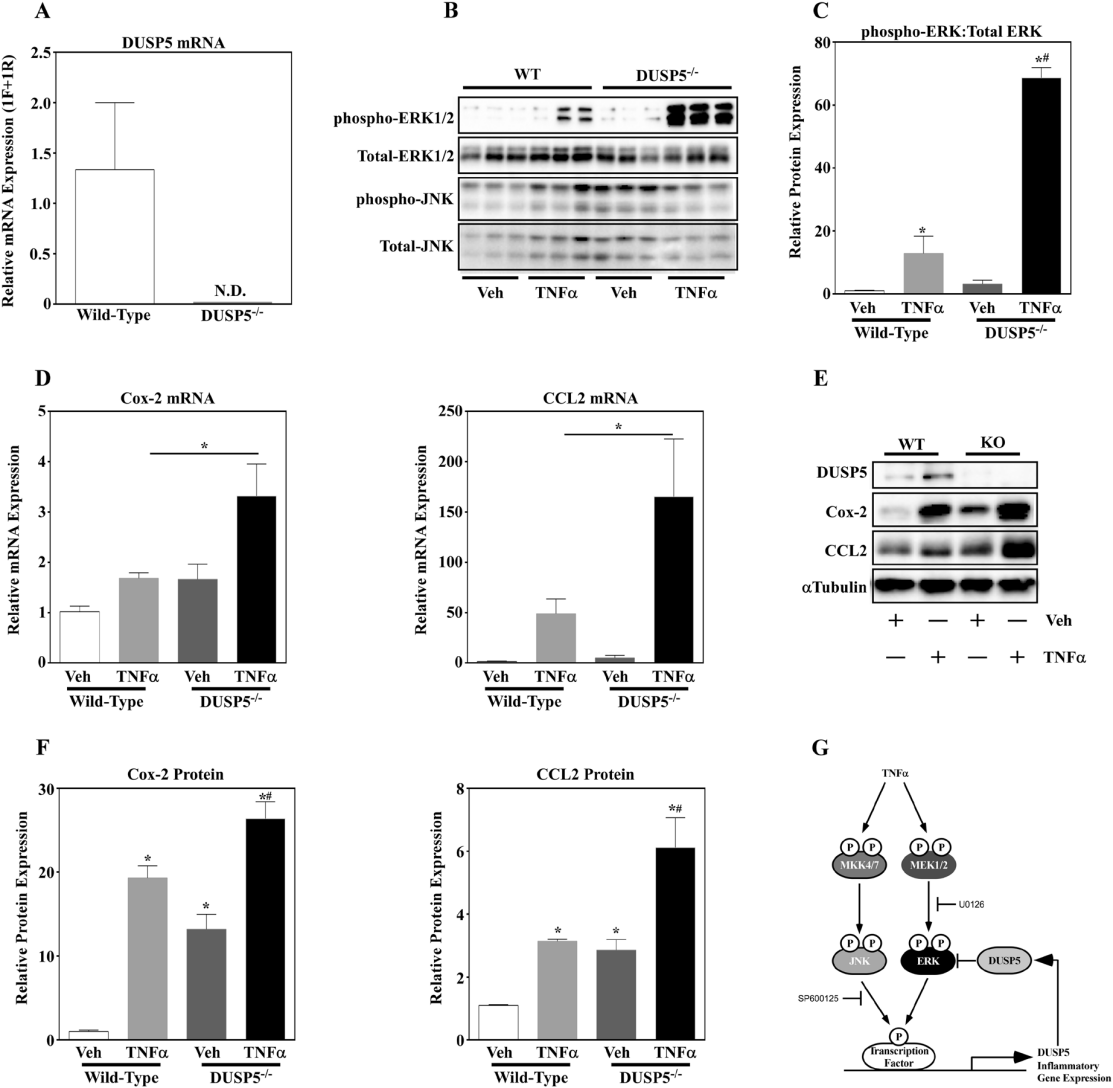
DUSP5 deletion exacerbates ERK phosphorylation and inflammatory gene expression *in vivo*. (**A**) RNA was isolated from Wild-type (WT) and DUSP5 knockout (DUSP^−/−^) mice and DUSP5 mRNA expression assessed via qPCR. WT and DUSP5 knockout mice were treated with vehicle (PBS + 0.1% BSA) or 1.25 µg TNFα for 1hr prior to sacrifice. (**B**) Protein was collected from white adipose tissue (WAT) and immunoblotted for phospho-ERK and JNK as well as total ERK and JNK. (**C**) Quantitation was assessed for ERK phosphorylation normalized to total ERK. (**D**) RNA was isolated from WAT and inflammatory gene expression of COX-2 and CCL2 examined via qPCR. (**E**) Protein from WT and knockout mice treated in the absence or presence of TNFα was assessed for DUSP5, Cox-2, CCL2 and αTubulin via immunoblotting and data for inflammatory protein expression (**F**) quantified via Image J software analysis. The collective data led to (**G**) a working model in which ERK- and JNK-dependent regulation of DUSP5 contributes to cross-talk and feedback inhibition of ERK phosphorylation and inflammatory gene expression in adipose tissue in response to TNFα.

To determine if DUSP5 regulates ERK phosphorylation and inflammatory gene expression in adipose tissue *in vivo*, 1.25 µg TNFα or vehicle control was administered by intraperitoneal (i.p.) injections to 10-week old, male DUSP5 null mice and wild-type (WT) littermates. Animals were sacrificed 1 hr post-treatment and white adipose tissue collected from epididymal fat pads. Total RNA was subsequently isolated. Primers were designed to amplify exon 2 and exon 3, consisting of the phosphatase catalytic domain, to demonstrate loss of the DUSP5 gene. Using these primers, amplification of DUSP5 was not detected in the WAT of DUSP5 null mice but was abundantly expressed in WT littermates (Fig. 5A). Protein was also isolated from WAT and immunoblotted for total and phosphorylated ERK and JNK. Consistent with 3T3-L1 adipocytes, ERK phosphorylation was significantly increased in DUSP5 null mice compared to WT littermates stimulated with TNFα (Fig. 5B,C). ERK phosphorylation trended higher with vehicle treatment in DUSP5 null mice compared to WT littermates although not significant. No differences were noted with total ERK or JNK, nor were differences noted in phosphorylated JNK. Based on these results, we next looked at inflammatory gene expression. Similar to the increase in ERK phosphorylation, Cox-2 and CCL2 was significantly increased in DUSP5 null mice compared to WT mice in response to TNFα (Fig. 5D). Moreover, loss of DUSP5 protein expression (Fig. 5E) led to a significant increase in Cox2 and CCL2 protein expression (Fig. 5E,F) in response to TNFα. Of note, basal expression of these two inflammatory molecules was also increased with loss of DUSP5 function. Complete immunoblots for *in vivo* data can be found in Supplemental Fig. 3.

## Discussion

The findings of this study demonstrate that DUSP5 acts as a negative regulator of adipocyte inflammatory gene expression that functions by regulating the magnitude and duration of ERK phosphorylation. We propose a model in which ERK and JNK-dependent induction of DUSP5 acts in a reciprocal feedback loop to regulate ERK signaling in a manner that links TNFα to pro-inflammatory gene expression in adipocytes (Fig. 5G).

Obesity is associated with chronic inflammation that is critical for the development of most obesity-mediated comorbidities including insulin resistance^12^. In response to metabolic stress, adipocytes produce and secrete chemokines (e.g. CCL-2/MCP-1) and cytokines (e.g. TNFα) that promote macrophage infiltration; this exacerbates adipose tissue inflammation and promotes insulin resistance^2,10,12^. We report that TNFα expression increased in adipose tissue dependent on the developmental stage of obesity (Fig. 1). As obesity increased TNFα expression increased suggesting infiltration of inflammatory immune cells and exacerbation of adipose tissue inflammation. Recent attention has been given to DUSPs, which have been shown to act as critical regulators of inflammation^14,22^. Similar to TNFα expression, DUSP5, an ERK1/2 specific phosphatase, increased with the development of obesity (Fig. 1). Furthermore, DUSP5 mRNA expression increased concomitant with increased TNFα expression (Fig. 1C), suggesting that TNFα regulates DUSP5 mRNA expression. Consistent with this hypothesis, we report that TNFα induced DUSP5 expression in adipocytes (Fig. 2D). These data suggest that DUSP5 acts as an ‘inducible’ endogenous regulator of ERK phosphorylation under conditions of obesity-mediated chronic, low-grade inflammation.

MAPKs such as ERK1/2 are essential regulators of adipose tissue inflammation, which link TNFα signaling to activation of downstream transcriptional programs that promote pro-inflammatory gene expression^3,7^. Indeed, genetic and pharmacological inhibition of ERK signaling has been shown to protect animals from diet-induced obesity, adipose tissue inflammation and insulin resistance^7,23,24^. We report that TNFα increased ERK1/2 phosphorylation in adipocytes (Fig. 2E). Moreover, we demonstrated that ERK activation was necessary for inflammation in adipocytes, where pharmacological inhibition of ERK1/2 attenuated inflammatory gene expression (i.e. IL-16, CCL2, and COX-2) (Fig. 3A).

MAPK-specific DUSPs have been shown to act as critical downstream regulators of MAPK activation and thus inflammation^14,22^. Our observations that ERK1/2 activation (i.e. phosphorylation) was transient (Fig. 2E), suggesting rapid deactivation i.e. dephosphorylation by phosphatases, support the postulate that phosphatases act as critical regulators of ERK signaling. Consistent with this postulate, induction of DUSP5 expression was inversely proportional to ERK1/2 phosphorylation in adipocytes in response to TNFα (Fig. 2D,E). DUSP5 has previously been implicated as a regulator of ERK1/2 signaling in response to pathological cardiac hypertrophy and fibrosis^21,25^. Indeed, we report that genetic loss of DUSP5 exacerbated ERK1/2 phosphorylation in adipocytes *in vitro* (Fig. 2G,H) as well as in adipose tissue *in vivo* (Fig. 5B,C).

Emerging literature demonstrates that DUSP5 is a critical regulator of inflammation and immune cell function^26,27^. Consistent with these reports, genetic loss of DUSP5 exacerbated TNFα-induced inflammatory gene expression in adipocytes (Fig. 3B) as well as inflammatory gene and protein expression in epididymal white adipose tissue (Fig. 5D,E and F). It should be noted that secretion of pro-inflammatory cytokines from preadipocytes, adipocytes as well as resident adipose tissue macrophages contribute to obesity-induced inflammation and its related comorbidities^2,12^. Thus, it would be important to delineate the role for DUSP5 in each cell type, as well as the impact of cellular cross-talk (e.g. adipocyte-macrophage) on DUSP5 regulation and function. While our *in vitro* data, suggests that loss of DUSP5 perpetuates inflammation in the adipocyte, we do not know how macrophage-adipocyte interactions might change this dynamic. While we can postulate that exacerbated adipose tissue inflammatory gene expression is a consequence of adipocyte signaling dysfunction (i.e. ERK1/2 phosphorylation), studies designed to knockdown DUSP5 in macrophages or tissue-specific knockout studies in mice would provide valuable insight into the actions of this phosphatase.

Canonical DUSP regulation involves feedback inhibition, in which MAPKs induce DUSPs that feedback inhibit MAPK phosphorylation^21,28^. Consistent with this notion, we report that DUSP5 is downstream of ERK1/2 and JNK activation, in which ERK or JNK inhibition attenuated TNFα-induced DUSP5 expression (Fig. 4). While pharmacological inhibitors can elicit off-target actions, it should be noted that others have reported that ERK1/2 regulates DUSP5 gene expression^21,29^. In addition, the use of independent compound inhibitors to target JNK and ERK for inhibition likely precludes off-target overlap and strongly supports the findings that JNK and ERK regulate DUSP5 message. Collectively therefore, these data suggest cross-talk between the JNK and ERK1/2 signaling cascades, in which JNK-mediated induction of DUSP5 can inhibit ERK1/2 phosphorylation. Others have reported similar cross-talk between signaling cascades, in which JNK-dependent induction of DUSP10/16 blocks ERK1/2 activation in COS-7 cells^30^. Together, these findings argue that intracellular signaling cross-talk is mediated, in part, by inducible DUSPs, such as DUSP5.

To conclude, our findings highlight the importance of DUSP5 as an endogenous regulator of ERK1/2 activation in adipocytes as well as regulation of inflammatory gene expression. Our data suggests that ERK1/2- and JNK-dependent regulation of DUSP5 creates cross-talk and/or feedback inhibition for ERK1/2 signaling in adipocytes in response to TNFα. While our findings suggest that DUSP5 potentially regulates ERK-mediated activation of obesity-induced inflammation, studies examining DUSP5 in obesity-mediated metabolic dysfunction have yet to be performed. Loss of DUSP5 function studies in rodent models of diet-induced obesity would highlight the role for this phosphatase in the regulation of ERK signaling, adipose tissue inflammation and insulin resistance. Studies outlined in our report examined the acute impact for DUSP5 loss of function on adipocyte ERK signaling and inflammatory gene expression, in which cells or mice were transiently exposed to inflammatory stimuli and in which DUSP5 responded to TNFα stimulation in a transient manner. As mice on a high fat diet had increased DUSP5 expression (Fig. 1) however, these data would suggest that long-term loss of DUSP5 function can greatly impact adipocyte function and metabolic outcomes.

## Methods

### Experimental animals

C57BL/6 J mice were rendered obese by diet. The supplier (Jackson Laboratories, Bar Harbor, Maine) housed and treated the mice until shipment 1 week prior to tissue harvest. Diet-induced obese C57BL/6 J mice were given free access to a high fat diet (HFD) consisting of 60% of total kcal from fat (Research Diets Inc. D12492) from 6 weeks of age, while lean controls were fed a low fat diet (LFD) consisting of 10% total kcal from fat (Research Diet Inc. D12450B). 10% kcal was from protein in both diets. The balance in caloric value was provided by differences in carbohydrate. Mice were given free access to food. Mice were shipped at 18 weeks and 24 weeks of age prior to euthanasia by CO_2_ gas asphyxiation. Epididymal white adipose tissue (WAT) was collected for total RNA.

Ten week-old, male, wild-type (WT) and DUSP5 null mice (sv/129 strain) were for TNFα experiments. WT and DUSP5 null animals were subjected to intraperitoneal (i.p.) injection with vehicle (PBS + 0.1% BSA) or 1.25 µg TNFα for 1 hr prior to asphyxiation, and WAT collected for protein and total RNA. Animal care and use was in compliance with the Institute of Laboratory Animal Research Guide for the Care and Use of Laboratory Animals and approved by the Institutional Animal Use and Care Committee at the University of Colorado Denver. All experiments were in accordance with the NIH guidelines.

### Cell culture

3T3-L1 preadipocytes were propagated in DMEM supplemented with 10% FBS until density-induced growth arrest. At 2 days post-confluence, growth medium was replaced with DMEM supplemented with 10% FBS, 0.5 mM 1-methyl-3-isobutylxanthine, 1 µM dexamethasone, and 1.7 µM insulin (MDI). Experiments were conducted in preadipocytes (d0) or mature adipocytes (d8) stimulated with 100 pM TNFα. All experiments were repeated at least 3 times.

### Immunoblotting

Cell monolayers were washed with PBS and scraped into ice-cold lysis buffer containing PBS (pH 7.4), 0.5% Triton X-100, 300 mM NaCl and protease/phosphatase inhibitor cocktail (Thermo Fisher). Cultured cells were sonicated prior to clarification by centrifugation. White adipose tissue was homogenized with the Next Advance Bullet Blender prior to clarification at 16,000 × g for 5 minutes. Protein concentrations were determined using a BCA Protein Assay Kit (Pierce). Proteins were resolved by SDS-polyacrylamide gel electrophoresis (PAGE), transferred to nitrocellulose membranes (BioRad). After transfer, membranes were blocked with 4% milk and probed with indicated primary antibodies for phospho-JNK (Cell Signaling Technology; 4668), phospho-pERK (Cell Signaling Technology; 4370), total JNK (Santa Cruz Biotechnology; sc-571), total ERK (Santa Cruz Biotechnology; sc-153), DUSP5 (Abcam; AB200708), phospho-c-Jun (Cell Signaling Technology; 9165), phospho-MAPKAPK2 (Cell Signaling Technology; 3007), MCP-1 (Cell Signaling Technology; 2029), or Cox-2 (Cell Signaling Technology; 12282). Horseradish Peroxidase (HRP)-conjugated secondary antibodies (Southern Biotech) were used at a concentration of 1:2000. SuperSignal West Pico chemiluminescence system (Thermo Scientific) and a ChemiDoc XRS + imager (BioRad) were used to detect protein. Densitometry was performed using Image J software and statistical analyses conducted via GraphPad Prism software.

### Real-Time qRT-PCR

Total RNA was isolated from WAT, preadipocytes or adipocytes via Qiazol (Qiagen) following the manufacturer’s protocol. Total RNA was quantified with a Nanodrop ND-1000 spectrophotometer and reverse-transcribed to cDNA using the Verso cDNA Synthesis Kit (Thermo Scientific; AB-1453). The reverse transcription (RT) master mix containing RT buffer, deoxyribonucleotide triphosphate (dNTP) mix, RT random primers, RNase inhibitor (1.0 U/µl), and RT enzyme was added to 500 ng RNA and RNase-free water.

PCR amplification was run using the BioRad CF96X qPCR instrument (BioRad) that consisted of enzyme activation at 95 °C for 15 minutes, followed by 40 cycles of denaturation at 95 °C for 15 sec combined with annealing at 55 °C for 30 sec and extension at 72 °C for 1 minute to examine DUSP5, CCL2, Cox-2 and IL-16 gene expression (Table 2). TNFα was examined using TaqMan^TM^ primers purchased from Applied Biosystems (ABI; Mm99999068_m1). Data were recorded and analyzed with Sequence Detector Software (BioRad) and graphs visualized with GraphPad Prism software. All data were presented as mean ± standard error of the mean (SEM). Data were normalized to 18 S, previously validated as a suitable reference gene^31^.

**Table 2:** Primer sequences used for qPCR in this study.

Genes of Interest	Forward Primer	Reverse Primer
Dusp5	5′-TCG CCT ACA GAC CAG CCT AT-3′	5′-GTA GTG TAG GTG GGT GGT GC-3′
CCL2	5′-CTC GGA CTG TGA TGC CTT AAT-3′ 5′-TGG ATC CAC ACC TTG CAT TTA-3′	5′-TGG ATC CAC ACC TTG CAT TTA-3′
COX-2	5′-CGA GTC GTT CTG CCA ATA GAA-3′	5′-CCT GGT CGG TTT GAT GTT ACT-3′
IL-16	5′-CAG GTC TCA AGA TGC CAA GT-3′	5′-TGG CTG CTG ATG GAG TAA AG-3′
**Reference Gene**		
18S	5′-GCCGCTAGAGGTGAAATTCTTG-3′	5′-CTTTCGCTCTGGTCCGTCTT-3′

### RNA Interference

Short interfering RNAs (siRNAs) for DUSP5 (Sigma; SASI_Mm01_00262935; DUSP5#1 and SASI_mM02_00300305; DUSP5#2) specific sequences as well as non-targeting siRNA control (siControl) sequences (MISSION siRNA Universal Negative Control #1; Sigma-SIC001) were transfected using Lipofectamine 3000 transfection reagent according to manufacturer’s (Thermo Scientific) protocol. Briefly, 3T3-L1 adipocytes were differentiated in 6-well culture dishes. Growth medium was then replaced with DMEM under serum-deprived conditions prior to addition of 3 µl of Lipofectamine 3000 reagent and either 100 nM DUSP5 specific siRNAs or non-targeting siRNA control for 3 hr. Growth medium was subsequently switched to fresh growth medium with FBS for 48 hr incubation prior to stimulation with TNFα. Cells lysate was harvested for RNA or protein as described above.

### Statistical analyses

Statistical analysis was completed by ANOVA followed by post-hoc testing (Tukey’s test was used for post-hoc analysis unless otherwise noted) using GraphPad Prism software. Statistical significance (defined as p < 0.05) is reported as applicable.

### Data Availability

Data will be made freely available upon request.

## Electronic supplementary material

Supplemental Information

## References

[1] Haslam, D. W. & James, W. P. Obesity. *Lance*t **36**6, 1197–1209, doi:S0140-6736(05)67483-1 [pii]; 10.1016/S0140-6736(05)67483-1 (2005).

[2] Gregor MF, Hotamisligil GS (2011). Inflammatory mechanisms in obesity. Annu Rev Immunol.

[3] Guilherme A, Virbasius JV, Puri V, Czech MP (2008). Adipocyte dysfunctions linking obesity to insulin resistance and type 2 diabetes. Nat Rev Mol Cell Biol.

[4] Solinas G, Becattini B (2017). JNK at the crossroad of obesity, insulin resistance, and cell stress response. Mol Metab.

[5] Hirosumi J (2002). A central role for JNK in obesity and insulin resistance. Nature.

[6] Zhang Y, Dong C (2007). Regulatory mechanisms of mitogen-activated kinase signaling. Cell Mol Life Sci.

[7] Fujishiro M (2003). Three mitogen-activated protein kinases inhibit insulin signaling by different mechanisms in 3T3-L1 adipocytes. Mol Endocrinol.

[8] Bost, F. *et al*. The extracellular signal-regulated kinase isoform ERK1 is specifically required for *in vitro* and *in vivo* adipogenesis. *Diabetes* **54**, 402–411, doi:54/2/402 [pii] (2005). 10.2337/diabetes.54.2.402 15677498

[9] Uysal KT, Wiesbrock SM, Marino MW, Hotamisligil GS (1997). Protection from obesity-induced insulin resistance in mice lacking TNF-alpha function. Nature.

[10] Hotamisligil GS, Shargill NS, Spiegelman BM (1993). Adipose expression of tumor necrosis factor-alpha: direct role in obesity-linked insulin resistance. Science.

[11] Romanatto T (2009). Deletion of tumor necrosis factor-alpha receptor 1 (TNFR1) protects against diet-induced obesity by means of increased thermogenesis. J Biol Chem.

[12] Hotamisligil GS (2017). Inflammation, metaflammation and immunometabolic disorders. Nature.

[13] Pearson G (2001). Mitogen-activated protein (MAP) kinase pathways: regulation and physiological functions. Endocr. Rev.

[14] Jeffrey KL, Camps M, Rommel C, Mackay CR (2007). Targeting dual-specificity phosphatases: manipulating MAP kinase signalling and immune responses. Nat Rev Drug Discov.

[15] Xu, H. *et al*. Dual specificity mitogen-activated protein (MAP) kinase phosphatase-4 plays a potential role in insulin resistance. *J. Biol. Chem* 278, 30187–30192, 10.1074/jbc.M302010200 [doi];M302010200 [pii] (2003). 10.1074/jbc.M302010200 12777378

[16] Wu, J. J. *et al*. Mice lacking MAP kinase phosphatase-1 have enhanced MAP kinase activity and resistance to diet-induced obesity. *Cell Metab* **4**, 61–73, doi:S1550-4131(06)00201-4 [pii]; 10.1016/j.cmet.2006.05.010 (2006). 10.1016/j.cmet.2006.05.010 16814733

[17] Rushworth LK (2014). Dual-specificity phosphatase 5 regulates nuclear ERK activity and suppresses skin cancer by inhibiting mutant Harvey-Ras (HRasQ61L)-driven SerpinB2 expression. Proc Natl Acad Sci USA.

[18] Mandl M, Slack DN, Keyse SM (2005). Specific inactivation and nuclear anchoring of extracellular signal-regulated kinase 2 by the inducible dual-specificity protein phosphatase DUSP5. Mol Cell Biol.

[19] Kidger AM (2017). Dual-specificity phosphatase 5 controls the localized inhibition, propagation, and transforming potential of ERK signaling. Proc Natl Acad Sci USA.

[20] Keyse SM, Dual-specificity MAP (2008). kinase phosphatases (MKPs) and cancer. Cancer Metastasis Rev.

[21] Ferguson BS (2013). Signal-dependent repression of DUSP5 by class I HDACs controls nuclear ERK activity and cardiomyocyte hypertrophy. Proc Natl Acad Sci USA.

[22] Zhang Y (2004). Regulation of innate and adaptive immune responses by MAP kinase phosphatase 5. Nature.

[23] Jager J (2011). Deficiency in the extracellular signal-regulated kinase 1 (ERK1) protects leptin-deficient mice from insulin resistance without affecting obesity. Diabetologia.

[24] Ozaki KI (2016). Targeting the ERK signaling pathway as a potential treatment for insulin resistance and type 2 diabetes. Am J Physiol Endocrinol Metab.

[25] Tao H (2016). Long noncoding RNA H19 controls DUSP5/ERK1/2 axis in cardiac fibroblast proliferation and fibrosis. Cardiovasc Pathol.

[26] Kutty RG (2016). Dual Specificity Phosphatase 5 Is Essential for T Cell Survival. PLoS One.

[27] Holmes DA, Yeh JH, Yan D, Xu M, Chan AC (2015). Dusp5 negatively regulates IL-33-mediated eosinophil survival and function. EMBO J.

[28] Keyse SM, Dual-specificity MAP (2008). kinase phosphatases (MKPs) and cancer. Cancer Metastasis Rev.

[29] Buffet C (2015). Dual Specificity Phosphatase 5, a Specific Negative Regulator of ERK Signaling, Is Induced by Serum Response Factor and Elk-1 Transcription Factor. PLoS One.

[30] Shen YH (2003). Cross-talk between JNK/SAPK and ERK/MAPK pathways: sustained activation of JNK blocks ERK activation by mitogenic factors. J Biol Chem.

[31] Ferguson BS, Nam H, Hopkins RG, Morrison RF (2010). Impact of reference gene selection for target gene normalization on experimental outcome using real-time qRT-PCR in adipocytes. PLoS. One.

