# Supplementary material for: DUSP5 functions as a feedback regulator of TNFα-induced ERK1/2 dephosphorylation and inflammatory gene expression in adipocytes
Source: Sci Rep. 2017 Oct 10;7:12879. doi: 10.1038/s41598-017-12861-y (PMC5635013; doi:10.1038/s41598-017-12861-y)
Supplement: Supplementary file 1 — Supplemental Information [file 41598_2017_12861_MOESM1_ESM.pdf]

Supplemental Information

DUSP5 functions as a feedback regulator of TNFα-induced ERK1/2 dephosphorylation and inflammatory gene expression in adipocytes

Justine S. Habibian[1], Mitra Jefic[1], Rushita A. Bagchi[2], Robert H. Lane[3], Robert A. McKnight[4], Timothy A. McKinsey[2], Ron Morrison[5*], Bradley S. Ferguson[1*]

[1] University of Nevada, Department of Agriculture, Nutrition, and Veterinary Sciences, Reno, Reno, Nevada, 89557, USA
[2] University of Colorado Denver-Anschutz Medical Campus, Department of Medicine, Division of Cardiology and Consortium for Fibrosis Research & Translation, Aurora, Colorado, 80045, USA
[3] Medical College of Wisconsin, Department of Pediatrics, Milwaukee, Wisconsin, 53226, USA
[4] University of Utah, Department of Pediatrics, Salt Lake City, Utah, 84108, USA
[5] University of North Carolina Greensboro, Department of Nutrition, Greensboro, North Carolina, 27412, USA
**corresponding authors: Bradley S. Ferguson, bferguson@unr.edu; Ron R Morrison, rfmorris@uncg.edu*

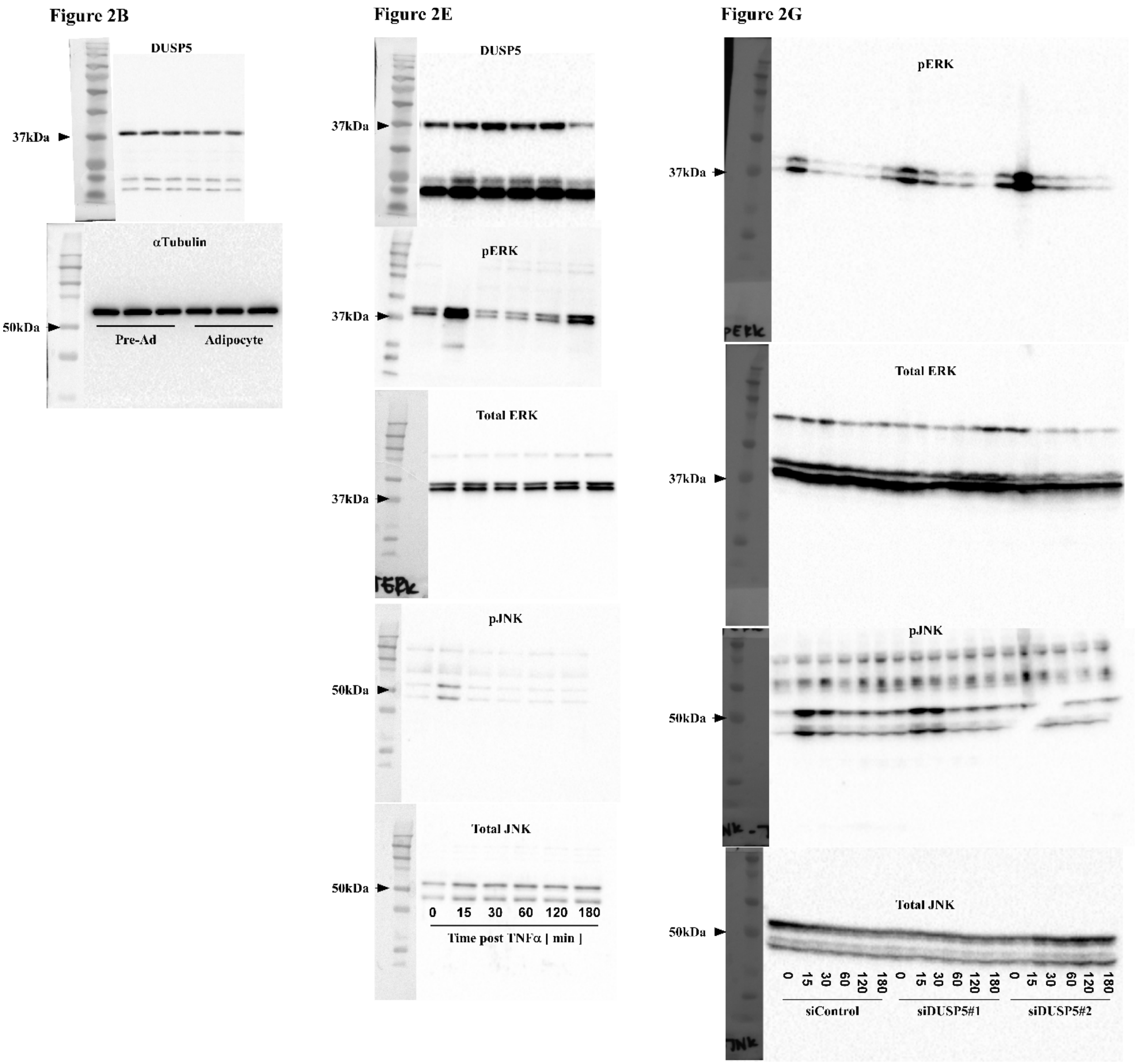

Supplementary Figure S1. Uncropped images from Figure 2B, E & G. All immunoblots were visualized via ChemiDoc XRS+ imager (BioRad). Densitometry was performed using Image J software and statistical analyses conducted via GraphPad Prism software.

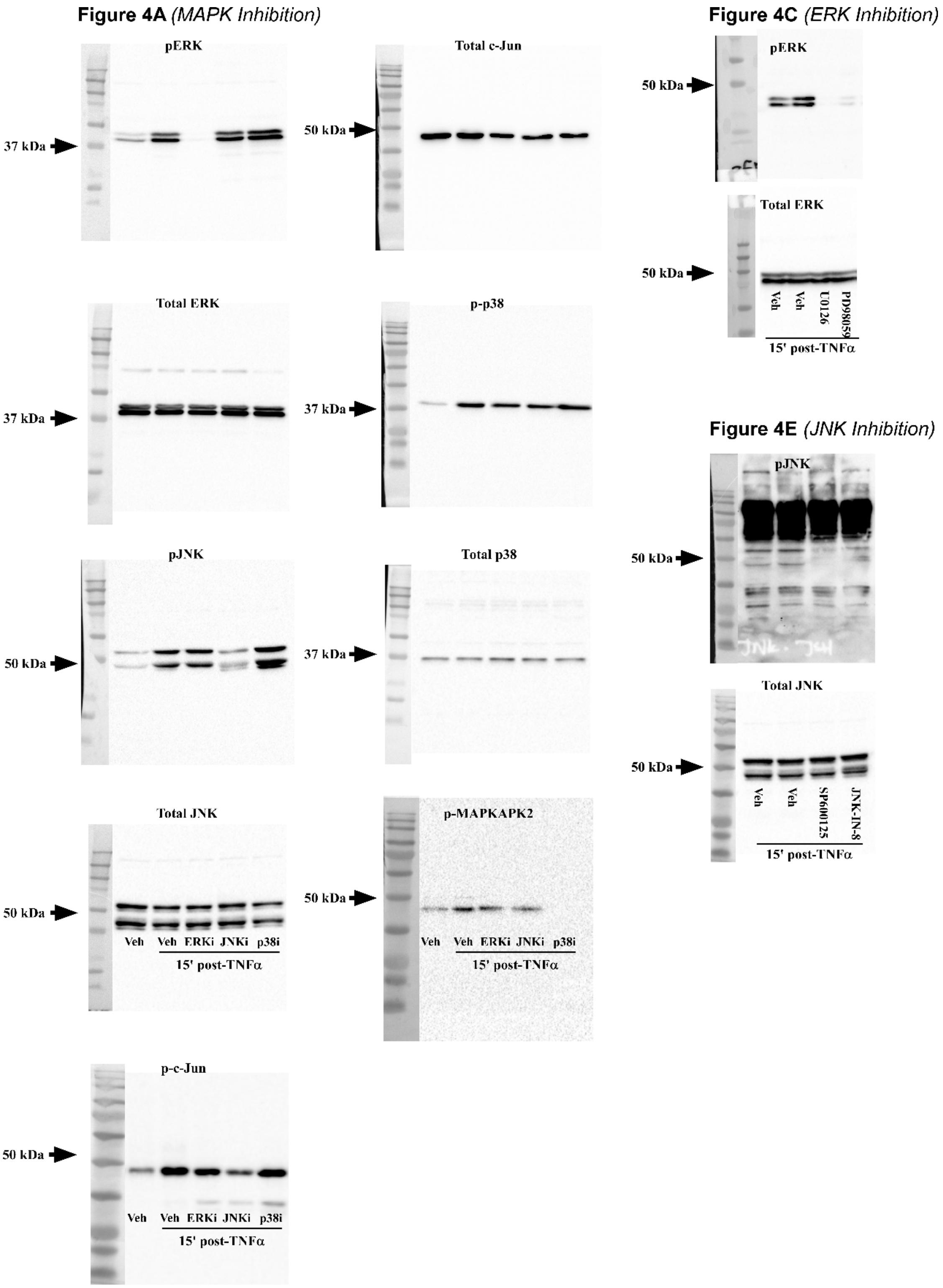

Supplemental Figure S2. Uncropped immunoblots of Figure 4A, C & E. All immunoblots were visualized via ChemiDoc XRS+ imager (BioRad). Densitometry was performed using Image J software and statistical analyses conducted via GraphPad Prism software.

Figure 5B

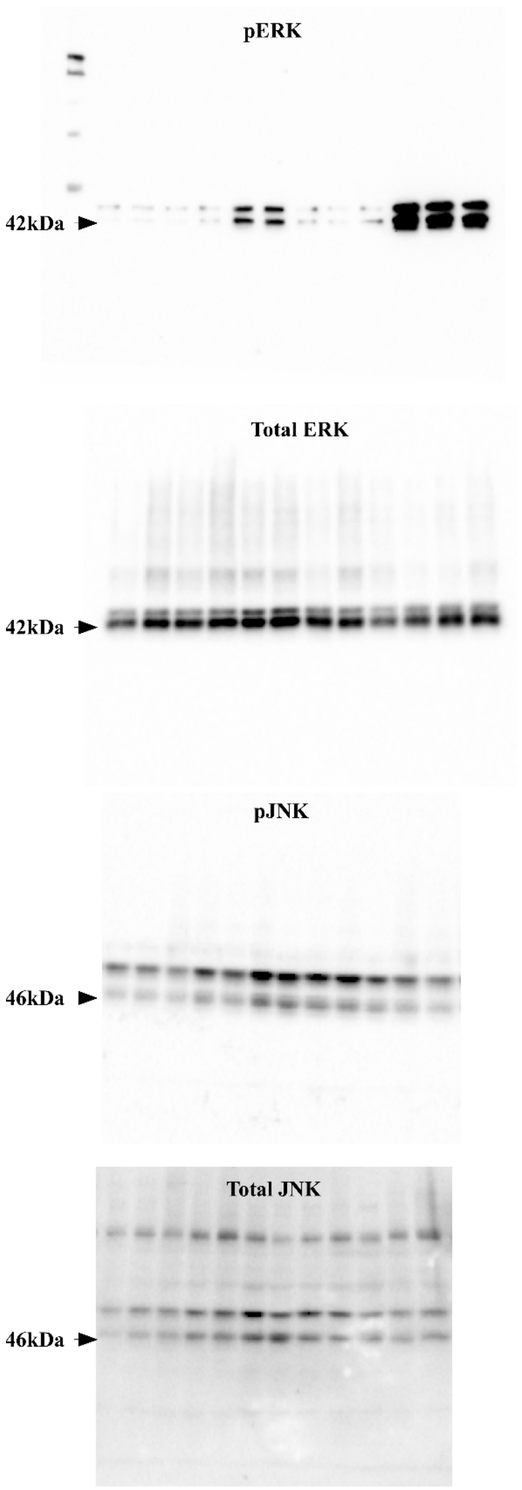

Figure 5E

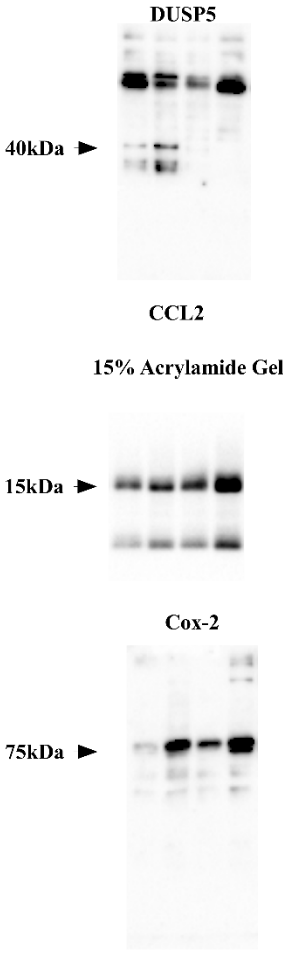

Supplemental Figure S3. Uncropped immunoblots of Figure 5B & E. All immunoblots were visualized via ChemiDoc XRS+ imager (BioRad). Densitometry was performed using Image J software and statistical analyses conducted via GraphPad Prism software.
